# Maternal and fetal outcomes of cesarean delivery and factors associated with its unfavorable management outcomes; in Ayder Specialized Comprehensive Hospital, Mekelle, Tigray, Ethiopia, 2017

**DOI:** 10.1186/s13104-019-4690-5

**Published:** 2019-10-07

**Authors:** Meresa Berwo Mengesha, Hadgay Hagos Adhanu, Desta Abraha Weldegeorges, Natnael Etsay Assefa, Weldu Mammo Werid, Mulu Gebretsadik Weldemariam, Fissaha Tekulu Welay, Hagos Degefa Hidru, Tesfay Tsegay Gebru

**Affiliations:** 1 Department of Midwifery, College of Medicine and Health Sciences, Adigrat University, P.O.BOX NO-50, Adigrat, Tigray Ethiopia; 2 Department of Midwifery, College of Medicine and Health Sciences, Mekelle University, Mekelle, Tigray Ethiopia; 3 Department of Public Health, College of Medicine Health Science, Adigrat University, Adigrat, Tigray Ethiopia; 4 Department of Nursing, College of Medicine and Health Science, Adigrat University, Adigrat, Tigray Ethiopia

**Keywords:** Cesarean delivery, Fetal outcomes, Maternal outcomes

## Abstract

**Objectives:**

This study aimed to determine the unfavorable outcomes and to assess factors contribute to the unfavorable management outcomes after cesarean deliveries in Ayder Specialized Comprehensive Hospital, Mekelle, Tigray, Ethiopia, 2017.

**Results:**

The unfavorable maternal management outcomes were Adhesion 28 (8.3%), excessive blood loss and blood transfusion 19 (5.6%), cesarean hysterectomy 10 (3%), relaparotomy 5 (1.5%), wound infection and wound dehiscence 23 (6.8%). Unfavorable fetal outcomes were were stillbirth 9 (2.6%), early neonatal death 8 (2.4%), low birth weight 58 (17.2%). women who did not book for Antenatal Care and having a history of previous cesarean delivery were found to be associated with unfavorable maternal outcomes and indications of cesarean delivery as obstructed labor was associated with unfavorable fetal outcomes.

## Introduction

Cesarean delivery is a surgical operation to deliver a fetus weighing greater than or equal to 1000 g or gestational age (GA) greater than or equal to 28 weeks in the Ethiopian context and 20 weeks in developed countries, through an incision on the anterior abdominal wall and the uterus [1, 2].

Increasing rate and number of cesarean deliveries are known to be associated with maternal risks (peripheral organ damage, bleeding, need for intensive care, long surgery time, hysterectomy and maternal death) [3–5]. Increasing rate and number of cesarean deliveries are known to be associated with fetal risks [prematurity, low APGAR (appearance, pulse, grimace, activity, respiration) score, stillbirth and early neonatal death] [3, 6, 7].

Though timing of the birth, the surgeon’s experience, the competence of the center, the surgical technique, and the risk of anesthesia are factors that play important roles in the emergence of complications, little is known to the factors contributing to the management outcomes in spite of the complications escalating [3, 8–11].

Moreover, many countries try to solve the problem by offering a trial of labor after caesarean delivery (TOLAC), reducing the number of primary CS by strict follow up, with appropriate indication and using instrumental deliveries [3, 12]. However, these efforts were not enough to minimize the complications.

Up to knowledge of Investigators, there is no study conducted in Tigray particularly in the study area and little is known about the factors associated with the unfavorable management outcomes.

## Main text

### Methodology

#### Study design, period and participants

Cross-sectional study was conducted on retrospective data recorded from November 2017 to January 2018 on cards of mothers who gave birth by caesarean delivery during the last 3 years (from September 8/2014 to September 8/2017).

#### Sample size and sampling technique

The sample size was calculated by a single population proportion formula by considering a 27.6% proportion of cesarean delivery prevalence, a 5% marginal error and a confidence interval of 95%. With the addition of a 10% contingency of incomplete cards, the yielded sample size was 338. Cards of mothers were selected using simple random sampling techniques.

#### Data collection procedure and instruments

A pretested checklist was used for collecting the data. The exposure variables which were studied in this research were: socio-demographic variables, obstetrics information of the women such as ANC booking status, duration of labor; mode of delivery, indication of CS, obstetric and medical conditions related to pregnancy and maternal outcomes.

#### Variables

*Dependent variables* Maternal management outcomes, fetal management outcomes.

*Independent variables* Maternal socio demographic characteristics, maternal obstetric characteristics, medical conditions related to pregnancy.

#### Data analysis and processing

The collected data was entered into epi info version 3.5.1 exported and, cleaned, processed and analyzed using SPSS version 20 statistical software descriptive analysis was made and the result was presented using mean, graphs, table of frequencies and narrative texts. Bivariate and multivariable analysis was made to assess the factors associated with maternal and fetal outcomes. The result was presented using the odds ratio with its 95% CI. Significance was declared at P-value less than or equal to 0.2 for bivariate logistic regression and P-value less than or equal to 0.05 for multivariate logistic regression and Presented using narrative text and tables.

#### Operational definitions

*Unfavorable maternal management outcomes* Presence of maternal complications at least one minor (extension of uterine incision, thin out of the lower uterine segment, a long stay of surgery time, etc.) or major complications (adhesion, uterine rupture, Caesarean Hysterectomy, wound dehiscence and others) after Caesarean delivery.

*Unfavorable fetal management outcomes* The presence of at least one of them (low birth weight, low APGAR score, early neonatal death, stillbirth).

#### Ethical issues

Ethical clearance was obtained from the institutional review board (IRB) of Mekelle university college of health sciences. Permission from Ayder Specialized Comprehensivehospital was obtained before the data collection started and data collectors were accompanied by an official letter from the Mekelle University, IRB and Ayder referral Hospital.

### Results

#### Socio-demographic characteristics of mothers who gave birth by cesarean delivery including their parity and ANC follow up

The median age of the mothers were 27 with IQR of 7. Most of the mothers were between 20 and 34 years 264 (78.1%) and from the total number of mothers who undergo cesarean delivery, 128 (37.9%) of the mothers are primipara, 182 (53.8%) between Para two and Para four. Eighteen (5.3%) of mothers with C/s were not booked for ANC in any health institution. Two hundred twenty-nine (67.8%) of mothers who had CS were referred from other institutions (for detail see Additional file 1: Table S1).

#### Characteristics of mothers who had previous CS

The numbers of mothers who had primary CS were 253 (74.9%), whereas 54 (16.1%) were repeated CS. From those who had repeated cesarean delivery 19 (47.5%) of them develop unfavorable maternal management outcomes and from those who tried trial of labor after cesarean delivery for vaginal delivery 35 (87.5) develop unfavorable maternal management outcomes and it was unsuccessful for vaginal delivery. Eighty-five (25.1%) of the mothers had history of previous caesarean delivery, out of these 20 (25%) had successful VBAC for detail see (Additional file 1: Table S2).

#### Maternal management outcomes with unfavorable outcomes

**Fig. 1:**
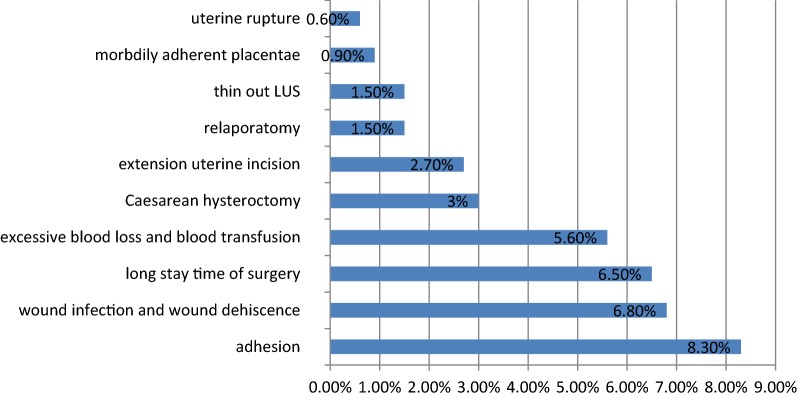
Shows unfavorable major and minor maternal complications after CS at Ayder Specialized Comprehensive Hospital from September 8, 2014, to September 8, 2017, (n = 338)

Adhesion including minimal and dense adhesion 28 (8.3%), excessive blood loss 19 (5.6%), cesarean hysterectomy 10 (3%), relaparotomy 5 (1.5%), were the major complications. Wound infection and dehiscence 23 (6.8%), long stay time of surgery 19 (6.5%), were found as minor maternal complications Fig. 1.

#### Fetal management outcome with unfavorable management outcomes

**Fig. 2:**
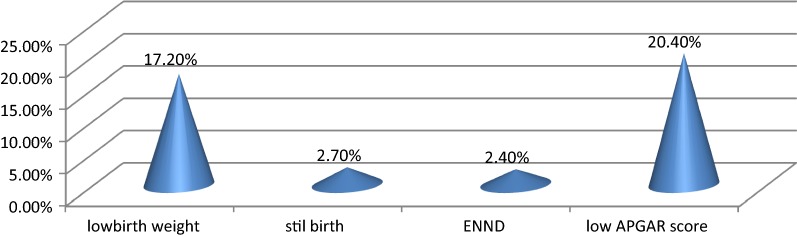
Shows unfavorable fetal outcomes after cesarean delivery at Ayder Specialized Comprehensive Hospital from September 8, 2014, to September 8, 2017, (n = 338)

Stillbirth 9 (2.6%), early neonatal death 8 (2.4%), low birth weight 58 (17.2%), and low APGAR score 69 (20.4%) were reported as fetal complications after cesarean delivery for detail see (Fig. 2).

#### Factors associated with maternal management outcomes

In bivariate logistic regression, referral status, ANC booking status, residency, gestational age in weeks, presence of previous CS, medical and obstetric conditions and indications for emergency CS, showed an association with maternal management outcome of CS at a P value of ≤ 0.2.

In multivariate logistic regression, ANC booking status and presence of previous CS were significantly associated (P value ≤ 0.05) with maternal management outcome of CS.

ANC follow up status had a significant association with unfavorable maternal management outcomes. Mothers who didn’t book for ANC were 9.6 times more likely to develop unfavorable maternal management outcome of CS compared to those who had ANC follow up during their pregnancy (AOR = 9.6, 95% CI (3.09, 38.9)). The Presence of previous CS had significant association with unfavorable management outcomes. Mothers who didn’t have previous history of cesarean delivery were 57% less likely to develop unfavorable maternal management outcomes compared to those mothers who had a history of previous CS (AOR = 0.43, 95% CI (0.43 (0.192, 0.95))) see on (Table 1).

**Table 1:** Bivariate and multivariate logistic regression results for factors associated with maternal management outcome of caesarean delivery at Ayder Specialized Comprehensive Hospital, from September 8, 2014 to September 8, 2017, (n = 338)

Independent variables	Number (%)	Unfavorable management outcome no (%)	Favorable management outcome no (%)	COR (95% CI)	AOR (95% CI)
Referral status					
Yes	229 (67.8%)	74 (21.9%)	155 (45.6%)	1.6 (0.9, 2.7)	
No	109 (32.2%)	25 (7.4%)	84 (24.9%)	1	1
ANC booking status					
Booked	320 (94.7%)	85 (25.1%)	235 (69.5%)	1	1
Un booked	18 (5.3%)	14 (4.1)	4 (1.1%)	9.6 (3.1, 30.2)	9.6 (3.1, 38.9)***
Residency					
Urban	196 (57.9%)	47 (13.9%)	149 (44.1%)	1	1
Rural	142 (42%)	52 (15.4%)	90 (26.6%)	1.83 (1.1, 2.9)	
Gestational age in weeks					
28–37	142 (42%)	52 (15.4%)	90 (26.6%)	0.6 (0.4, 0.9)	
37–42	193 (57.1%)	45 (13.3%)	148 (43.8%)	0.3 (0.26, 3.3)	
≥ 42	3 (0.9%)	2 (0.6%)	1 (0.3%)	1	1
Presence of previous CS					
Yes	85 (25.1%)	39 (11.5%)	46 (13.6%)	1	1
No	253 (74.9%)	60 (17.8%)	193 (57.1%)	0.4 (0.2, 0.6)	0.43 (0.2, 0.9)***
Medical and obstetric conditions					
Yes	73 (21.6%)	32 (9.5%)	41 (12.1%)	0.43 (0.3, 0.7)	
No	265 (78.4%)	67 (19.8%)	198 (58.6%)	1	1
Indications of EMRCS					
CPD	56 (16.6%)	10 (2.96%)	46 (13.6%)	0.26 (0.1, 0.8)	
NRFHP	86 (25.4%)	12 (3.6%)	74 (21.9%)	0.2 (0.1, 0.6)	
Obstructed labor	20 (5.9%)	15 (4.4)	5 (1.8%)	3.6 (0.95, 14.02)	
Malpresentations	12 (3.6%)	2 (0.6)	10 (2.97%)	1	1

*COR* crude odds ratio, *AOR* adjusted odds ratio

*** Significant variable in multivariate

#### Factors associated with fetal management outcome

In bivariate logistic regression, referral status, maternal management outcome, surgeries performed by, duration of labor, presence of previous CS, medical and obstetric conditions and indications for emergency CS, showed association with unfavorable fetal management outcome at a P value of ≤ 0.2.

In multivariate logistic regression, an indication of CS as preeclampsia/eclampsia and obstructed labor were significantly associated (P value ≤ 0.05) with unfavorable fetal management outcomes.

Indication of emergency CS as obstructed labor had a great significant association with unfavorable fetal management outcomes. Mothers who had CS with indication of obstructed labor were 8.3 times more likely to develop unfavorable fetal management outcome than with indication of mal-presentations (AOR = 8.3, 95% CI (1.17, 60)) and indications as pre-eclampsia/eclampsia were 9.8 times more likely to develop unfavorable fetal management outcome than with indications as mal-presentation (AOR = 9.8, 95% CI (1.03, 94)) see in (Additional file 1: Table S3).

### Discussion

In this study, the most common major and minor maternal complications were Adhesion 28 (8.3%), excessive blood loss 19 (5.6%), cesarean hysterectomy 10 (3%), relaparotomy 5 (1.5%), morbidly adherent placentae 3 (0.9%). This finding is slightly lower than study done in Adiyaman teaching hospital, Southeast Turkey [3], but, in line with the studies done in lady Aitchison hospital, Lahore Pakistan [6], Haifa, Israel [13], Shalamar hospital, Lahore Pakistan [14], Jimma hospital, Ethiopia [15]. The difference with the first study might be, the study was conducted in those who had multiple repeat CS.

Wound infection and dehiscence 23 (6.8%), long stay time of surgery 19 (6.5%), extension of uterine incision 9 (2.7%) and thin out LUS 5 (1.5%) were found as minor maternal complications, which is consistent with the studies done in Al Qassimi hospital, UAE [9], Shalamar hospital, Lahore Pakistan [14], Jimma hospital, Ethiopia [15]. Despite the full coverage of antibiotics, surgical site infection was common this might be due to patient factor, surgeon factor, and environmental factors.

The Finding of this study revealed that Unfavorable fetal outcomes, stillbirth 9 (2.6%), early neonatal death 8 (2.4%), low birth weight 58 (17.2%), low APGAR score 69 (20.4%) were reported as fetal complications after cesarean delivery, this finding is in line with findings found in northwest Nigeria [16], Jimma, south west Ethiopia [6], Atat Hospital, Gurage zone Ethiopia [8]. This might be due to a lot of mothers with obstetric and medical conditions undergo caesarean delivery without checking fetal lung maturity to save the life of mother.

This study showed that Mothers who didn’t book for ANC were 9.6 times more likely to develop unfavorable maternal management outcomes of CS compared to those who had ANC follow up during their pregnancy (AOR = 9.6, 95% CI (3.09, 38.9)). This study is similar to the study done in Atat hospital [8], Gurage and Tanzania teaching hospital [17]. This is because antenatal visits of the pregnant mother are very important as they provide a chance of receiving information about their mode of delivery after caesarean delivery, scheduled time, place of delivery, screening for malpresentations, screening for preeclampsia, screening for congenital anomalies, assessing mothers who needs special care and frequent visit.

Finding of this study also showed that mothers who didn’t had previous history of caesarean delivery were 57% less likely to develop unfavorable maternal management outcome compared to those mothers who had a history of previous CS (AOR = 0.43, 95% CI (0.43 (0.192, 0.95))). This is consistent with the study done in lady Aitchson hospital, Lahore Pakistan [6], Al Qassimi hospital, UAE [9]. This might be increased cesarean delivery with the limited option of TOLAC and due to poor surgical skill of a surgeon.

This study revealed that mothers who had CS with an indication of obstructed labor were 8.3 times more likely to develop unfavorable fetal management outcomes than with indication of mal-presentations (AOR = 8.3 95% CI (1.17, 60)). This finding is in line with the study done in Northwest Nigeria [16], but in contrary with study done in Ethiopia; caesarean delivery at the national level [14], indications of CS as malpresentation and fetal distress was associated with poor fetal outcome. This might be due to differences in hospital management of cases like malpresentation (breech presentation), fetal distress, and referral status.

Indications as pre-eclampsia/eclampsia were 9.8 times more likely to develop unfavorable fetal management outcome than with indications as mal-presentation (AOR = 9.8, 95% CI (1.03, 94)). This study is in line with the study done in Nigeria [16]. This might be because of the procedure is done for maternal indication, neglecting fetal conditions.

## Conclusions and recommendations

Finding of this study showed that adhesion, excessive blood loss, caesarean hysterectomy, wound infection and dehiscence were the dreadful unfavorable maternal management outcomes. and this study found that, still birth, early neonatal death, and low birth weight were Unfavorable fetal outcomes. The finding of this study also showed that those mothers who had previous caesarean delivery and un-booked for ANC were associated with unfavorable Maternal management outcomes and their CS indications as prolonged obstructed labor and preeclampsia were associated with poor fetal outcomes.

For health professionals; we recommend that trial of labor after caesarean delivery (TOLC) should be encouraged in those who fulfill the criteria. The Utilization of antenatal care and proper antenatal counseling should be encouraged. Early referral and proper way of monitoring of labor by partograph should be encouraged. For researchers; needs further prospective study to identify predictors with management outcomes.

## Limitation of the study

- Full Information about neonatal complications was not found from mothers’ card or medical record profiles. Getting complete information regarding subsequent neonatal events was difficult. Therefore we were limited to focus only on immediate outcomes (alive/dead) and early neonatal complications.
- Retrospective nature of the study and lack of some data due to inappropriate and/or non- recording of certain variables.

## Supplementary information

**Additional file 1: Table S1.** Distribution of cesarean section cases by socio-demographic Characteristics, parity and antenatal care (ANC) follow up at Ayder Specialized Comprehensive Hospital, from September 8, 2014 to September 8, 2017, (n = 338). **Table S2.** Shows presence of successful VBAC and number of previous cesarean delivery of women who had history of previous caesarean delivery at Ayder Specialized Comprehensive Hospital, from September 8, 2014 to September 8, 2017, (n = 338). **Table S3.** Bivariate and Multivariate logistic regression results for factors associated with fetal management outcome of caesarean delivery at Ayder Specialized Comprehensive Hospital from September 8, 2014 to September 8, 2017, (n = 338).

## Data Availability

The datasets used and/or analyzed during this study are available from the corresponding author on reasonable request.

## Abbreviations

ANC: antenatal care
AOR: adjusted odds ratio
BTL: bilateral tubal ligation
CS: caesarean section
CPD: cephalo pelvic disproportion
EMRCS: emergency caesarean section
GA: gestational age
LUS: lower uterine segment
NRFHP: non reassuring fetal heart beat pattern
SPSS: statistical package for social science
TOLC: trial of labor after caesarean delivery
