# Supplementary material for: Maternal and fetal outcomes of cesarean delivery and factors associated with its unfavorable management outcomes; in Ayder Specialized Comprehensive Hospital, Mekelle, Tigray, Ethiopia, 2017
Source: BMC Res Notes. 2019 Oct 7;12:650. doi: 10.1186/s13104-019-4690-5 (PMC6781415; doi:10.1186/s13104-019-4690-5)
Supplement: Supplementary file 1 — Additional file 1: Table S1. Distribution of cesarean section cases by socio-demographic Characteristics, parity and antenatal care (ANC) follow up at Ayder Specialized Comprehensive Hospital, from September 8, 2014 to September 8, 2017, (n = 338). Table S2. Shows presence of successful VBAC and number of previous cesarean delivery of women who had history of previous caesarean delivery at Ayder Specialized Comprehensive Hospital, from September 8, 2014 to September 8, 2017, (n = 338). Table S3. Bivariate and Multivariate logistic regression results for factors associated with fetal management outcome of caesarean delivery at Ayder Specialized Comprehensive Hospital from September 8, 2014 to September 8, 2017, (n = 338). [file 13104_2019_4690_MOESM1_ESM.docx]

Table S1: Distribution of cesarean section cases by socio-demographic Characteristics, parity and antenatal care (ANC) follow up at Ayder Specialized Comprehensive Hospital, from September 8, 2014 to September 8, 2017, (n=338).

| Variables | | Frequency | | | Percentage (%) | |
| --- | --- | --- | --- | --- | --- | --- |
| Age (years) | | | | | | |
| <=19 | | 18 | | | 5.3 | |
| 20-34 | | 264 | | | 78.1 | |
| >=35 | | 56 | | | 16.6 | |
| Parity | | | | | | |
| Primipara | | | 128 | | 37.9 | |
| Para (2-4) | | | 182 | | 53.8 | |
| Grandmulitipara | | | 28 | | 8.3 | |
| ANC Booking status | | | | | | |
| Booked | | | 320 | | 94.7 | |
| Un booked | | | 18 | | 5.3 | |
| Ethnicity | | | | | | |
| Tigray | | | 330 | | 97.6 | |
| Afar | | | 7 | | 2.1 | |
| Amhara | | | 1 | | 0.3 | |
| Residence | | | | | | |
| Urban | | | 196 | | 58 | |
| Rural | | | 142 | | 42 | |
| Referral | | | | | | |
| Yes | | | 229 | | 67.8 | |
| No | | | 109 | | 32.2 | |

Table S2: shows presence of successful VBAC and number of previous cesarean delivery of women who had history of previous caesarean delivery at Ayder Specialized Comprehensive Hospital, from September 8, 2014 to September 8, 2017, (n=338).

| Variable | Category | Maternal Favorable management outcomes (%) | Maternal unfavorable management outcomes (%) | Total (%) |
| --- | --- | --- | --- | --- |
| Number of previous CS | Primary(1) | 38(84.4) | 18(45) | 56(65.9) |
|  | Repeated(2) | 7(15.6) | 19(47.5) | 26(30.6) |
|  | 3 and above | 0 | 3(7.5) | 3(3.5) |
| Presence of successful VBAC | No | 29(64.4) | 35(87.5) | 64(75.3) |
|  | Yes | 16(35.6) | 5(12.5) | 21(24.7) |
|  |  | Fetal favorable management outcomes | Fetal unfavorable management outcomes | Total |
| Number of previous CS | Primary(1) | 52(66.7) | 4(57.1) | 56(65.9) |
|  | Repeated(2) | 23(29.5) | 3(42.9) | 26(30.6) |
|  | 3 and above | 3(3.8) | 0 | 3(3.5) |
| Presence of successful VBAC | No | 57(24) | 7(87.5) | 64(75.3) |
|  | yes | 20(26) | 1(12.5) | 21() |

CS= Cesarean Section

VBAC= Vaginal Birth after Cesarean Delivery

Table S3: Bivariate and Multivariate logistic regression results for factors associated with fetal management outcome of caesarean delivery at Ayder Specialized Comprehensive Hospital from September 8, 2014 to September 8, 2017, (n= 338)

| Independent variables | Number (%) | Unfavorable management outcome No (%) | Favorable management outcome No (%) | COR (95% CI) | AOR (95%) CI |
| --- | --- | --- | --- | --- | --- |
| Referral status  Yes  No | 229(67.8%)  109(32.2%) | 59(17.5%)  13(3.84%) | 170(50.3%)  96(28.4%) | 2.6(1.3, 4.9)  1 | 1 |
| Maternal management outcome  Favorable  Unfavorable | 239(70.7%)  99(29.3%) | 34(10.5%)  38(11.24) | 205(60.7%)  61(18.04%) | 1  3.8(2.2, 6.5) | ­  1 |
| Surgeries performed by  Senior  Junior | 112(33.1%)  226(66.9%) | 15(4.4%)  57(16.7%) | 97(28.7%)  169(50%) | 1  2.2(1.17, 4.05) | 1 |
| Duration of labor  <12hr  12-24  >=24 | 131(38.8%)  107(31.7%)  5(1.5%) | 21(6.2%)  26(7.7%)  4(1.2%) | 110(32.5%)  81(23.9%)  1(0.3%) | 1  1.68(0.9, 3.2)  20.95(2.2, 196.9) | 1 |
| Presence of previous CS  Yes  No | 85(25.14%)  253(74.9%) | 7(2.1%)  65(19.2%) | 78(23%)  18(5.32%) | 1  0.26(0.1, 0.6) | 1 |
| Medicaland obstetric conditions  Yes  No | 73(21.6%)  265(78.4%) | 28(8.3%)  44(13.01%) | 45(13.3%)  221(65.4%) | 3.125(1.2, 5.5 )  1 | 1 |
| Indications of EMRCS  Preeclampsia/eclampsia  Obstructed labor  Malpresentations | 20(5.9%)  20(5.9%)  12(3.6%) | 2(0.6%)  14(4.1%)  2(0.6%) | 18(5.3%)  6(1.8 %)  10(2.95%) | 14(2.3, 90.5)  21(3.66, 120)  1 | 9.8(1.03, 94)**8.3(1.2, 60)***  1 |

COR= crude odds ratio, AOR= adjusted odds ratio, ***= significant variable in multivariate

EMRCS= Emergence Caesarean Section

CS= Caesarean Section
